# An Important Decision

**Published:** 1914-07

**Authors:** 


### An Important Decision.

The question of the State's right to interfere with one's personal liberty in the interest of the individual's welfare and the good of society is well exemplified by the following decision handed down by Judge Wilkins in favor of the Brooklyn Society for the Prevention of Cruelty to Children:

"The father and mother of four children had refused to have the children treated at the hospital for syphilis. Two of the children had had a Wasserman test, which was positive; the other two children were suspected of having syphilis, and the parents would not allow them to be examined by the Wasserman test. At the hearing, the testimony of four physicians, two bacteriologists, and four social workers was taken, and the family physician testified to the effect that the children would be best off if the court should commit the children to the care of the society, which he did. The health department authorities and physicians were willing to, and did, co-operate in the case. They were protected by subpoenas from the charge of disclosing confidential information. The basis for this decision rests on the civil law principle that the State may interfere with the parental custody when such intervention is necessary for the welfare of the children."

---

THE International Conference on Opium opened at The Hague on June 15th, when it was announced that all the States except Servia, Turkey, and Greece had given their adherence to the decisions of the last convention.

